# Research on Acoustic Scene Classification Based on Time–Frequency–Wavelet Fusion Network

**DOI:** 10.3390/s25133930

**Published:** 2025-06-24

**Authors:** Fengzheng Bi, Lidong Yang

**Affiliations:** 1 School of Digital and Intelligent Industry, Inner Mongolia University of Science and Technology, Baotou 014010, China; bfz@stu.imust.edu.cn; 2 Inner Mongolia Key Laboratory of Pattern Recognition and Intelligent Image Processing, Baotou 014010, China

**Keywords:** acoustic scene classification, wavelet transform, visual state space, KAN

## Abstract

Acoustic scene classification aims to recognize the scenes corresponding to sound signals in the environment, but audio differences from different cities and devices can affect the model’s accuracy. In this paper, a time–frequency–wavelet fusion network is proposed to improve model performance by focusing on three dimensions: the time dimension of the spectrogram, the frequency dimension, and the high- and low-frequency information extracted by a wavelet transform through a time–frequency–wavelet module. Multidimensional information was fused through the gated temporal–spatial attention unit, and the visual state space module was introduced to enhance the contextual modeling capability of audio sequences. In addition, Kolmogorov–Arnold network layers were used in place of multilayer perceptrons in the classifier part. The experimental results show that the proposed method achieves a 56.16% average accuracy on the TAU Urban Acoustic Scenes 2022 mobile development dataset, which is an improvement of 6.53% compared to the official baseline system. This performance improvement demonstrates the effectiveness of the model in complex scenarios. In addition, the accuracy of the proposed method on the UrbanSound8K dataset reached 97.60%, which is significantly better than the existing methods, further verifying the generalization ability of the proposed model in the acoustic scene classification task.

## 1. Introduction

Acoustic scene classification (ASC) is a multi-category classification task in audio signal processing that aims to identify and classify the environmental context or specific scene presented by a given audio signal, such as an airport, street or park [1]. Its core value is firstly reflected in the field of health and environmental protection, where the technique can accurately identify noise sources and provide a basis for noise pollution control, thus reducing the health hazards to residents [2]. In addition, the technology has been widely used in many fields such as public monitoring, smart home, and personalized environment perception services in wearable devices [3]. Therefore, improving the classification and generalization performance of the ASC model has important research significance and wide application prospects. As one of the core tasks in the Detection and Classification of Acoustic Scenes and Events (DCASE), ASC aims to achieve high classification accuracy and strong generalization capability, even under conditions of limited training data and diverse recording devices [4].

In recent years, the transformer model, with its unique encoder–decoder structure, multi-head attention mechanism, and positional encoding, has had an advantage in capturing the global context, but it still faces computational efficiency and memory consumption problems [5,6]. Therefore, the Mamba model, based on the state space model (SSM), introduces input selectivity parameters and features linear complexity as well as strong long-sequence modeling capabilities [7,8,9]. Its parallel selective scanning mechanism improves efficiency while capturing global contextual information. For multiple tasks, Mamba shows a comparable performance to that of the transformer model [10,11]. This study [12] proposes the use of Audio Mamba (AuM) to achieve a performance comparable to that of the audio spectrum transformer (AST) in ASC tasks. It should be noted that the transformer model and the AuM model were not used in this paper, and the relevant content was used only for the background to highlight the advantages of the Mamba model for sequence modeling.

In ASC, multilayer perceptrons (MLPs) are commonly used as classifiers; however, their fixed activation functions and linear weights limit their ability to model complex features. Recently, the Kolmogorov–Arnold network (KAN) has enhanced both the modeling capacity and interpretability of complex features by replacing traditional structures with learnable spline functions [13,14]. The experimental results presented in Section 3.1 demonstrate that the design of the KAN model effectively captures nonlinear time–frequency characteristics in audio data, thus improving the classification accuracy under multi-device conditions.

Recently, the authors of [15] introduced harmonic-shock source separation (HPSS) to separate time and frequency information from spectrograms and augmented convolutional neural network (CNN) to learn key time–frequency features, but the computational cost of the method is high. For this reason, the time–frequency separate network (TF-SepNet) [16] was designed based on the principle of deconvolutional entanglement to extract features from the dual path structure of the time and frequency dimensions, which reduced the computational complexity and improved the performance at the same time. Ref. [16] shows that the accuracy of the TF-SepNet is higher than that of conventional methods that use successive convolutional kernels (i.e., features are extracted simultaneously in the time and frequency dimensions) [17,18].

Currently, there are still challenges in the research on ASC tasks. First, the temporal continuity of audio signals results in dependencies between different time frames, and previous studies have shown that capturing both short-range and long-range dependencies is crucial for learning effective audio representations [11]. Secondly, there exists variability in audio data captured from different devices [19]. Finally, in deep learning, audio data are converted into the form of a spectrogram as the input, but only the convolution operation is performed on the spectrogram, which does not fully utilize the time-domain information, the frequency-domain information, or the high- and low-frequency information of the spectrogram [20].

This study aimed to improve the sequence modeling capability and classification performance of models for cross-device audio data in the ASC task. To achieve this, we propose a time–frequency–wavelet fusion network (TFWFNet), designed to fully exploit the information contained in spectrograms and enhance the modeling of contextual audio features. In addition, various data augmentation strategies were employed to increase the data diversity, mitigate overfitting, and improve the model’s generalization ability across different recording devices.

The main innovations of this study are as follows: First, a time–frequency–wavelet module (TFWM) was proposed, which integrates wavelet transform into the time–frequency fusion structure to enhance the model’s ability to extract informative features from spectrograms. Second, a gated temporal–spatial attention unit (GTSAU) is designed by combining temporal and spatial attention mechanisms with a gating strategy, thereby improving the model’s capacity to capture salient information. Third, a visual state space (VSS) module is introduced to strengthen the modeling of contextual information and spatial relationships in audio data. Finally, a classifier based on the KAN layer is constructed to further improve the model’s generalization performance under complex and diverse data distributions.

The uniqueness of this study lies in its innovative fusion strategy and structural design for multidimensional feature information. Specifically, it fuses spectrogram features from the time dimension, frequency dimension, and the high- and low-frequency paths extracted via wavelet transform, providing a richer feature representation for modeling in the ASC task. Furthermore, the introduction of the VSS module into the ASC task enables a deeper exploration of the structural relationships between spatio-temporal features and contextual information, thereby broadening the research perspective in this field. In addition, the incorporation of the KAN layer facilitates efficient modeling of complex nonlinear relationships.

## 2. Methods

**Figure 1:**
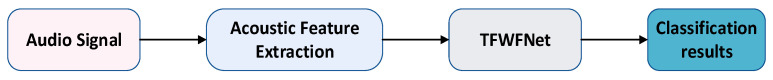
Schematic structure of the acoustic scene classification system.

Figure 1 illustrates the schematic structure of the overall ASC system, which mainly consists of three components: acoustic feature extraction, the proposed TFWFNet model, and the final classification results. The acoustic feature extraction module is responsible for capturing distinguishing features of various sound scenes from audio signals. Typically, the signals are converted into spectrograms using the short-time Fourier transform, which are then used as input to the subsequent network model.

**Figure 2:**
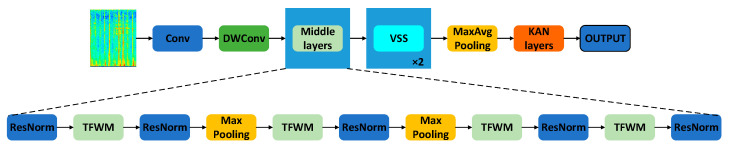
Time–frequency–wavelet fusion network. DWConv: depthwise convolution, VSS: visual state space, ResNorm: residual normalization, TFWM: time–frequency–wavelet module.

The TFWFNet designed in this study consists of three main components. The first component is the TFWM, which integrates spectrogram information across three dimensions: the time dimension, frequency dimension, and high- and low-frequency paths extracted via wavelet transform. It also incorporates the GTSAU, proposed in this paper, to fuse multidimensional features, thereby enhancing the performance and robustness of acoustic scene classification tasks. The second component is the VSS module, aimed at improving the model’s ability to capture contextual information and understand complex spatial relationships in acoustic scenes. The third component is a classifier composed of the KAN layers, designed to efficiently fit complex nonlinear relationships in the data and enhance generalization across heterogeneous sources. The overall architecture of the model is illustrated in Figure 2.

### 2.1. Data Augmentation

Data augmentation is a key technique in ASC tasks. By applying various transformations to audio signals during training, it increases the amount and diversity of training samples, thereby improving the model’s generalization ability and mitigating overfitting. In this study, a combination of MixUp [21], Freq MixStyle [22], SpecAugmentation [23], and Device Impulse Response (DIR) [24] Augmentation is employed to enhance data diversity.

Among them, DIR augmentation is performed by randomly selecting 1 of 66 device impulse responses to convolve with the audio signal, thereby effectively simulating audio captured by different devices and helping the model learn device-related variations.

### 2.2. Time–Frequency–Wavelet Module

Wavelet transform, as a multi-resolution analysis tool, is widely used in tasks such as decomposition, compression, and denoising. It enables more flexible and efficient extraction of high-frequency and low-frequency features. By applying wavelet transform, both local high-frequency detail features and global low-frequency trend features in spectrograms can be effectively captured. Moreover, wavelet transform not only mitigates feature interference across frequency components in spectrograms but also provides a more robust feature representation for ASC tasks.

However, different wavelets have different time–frequency localization characteristics and offer distinct advantages in extracting high- and low-frequency information from spectrograms. The Coif3 wavelet (Coiflet wavelet family, order 3) is a third-order wavelet in the Coiflet series, featuring symmetry and orthogonality. It efficiently balances the decomposition of high- and low-frequency components, provides clear time–frequency features, reduces information redundancy, and offers high computational efficiency. In contrast, the Haar wavelet is the simplest compactly supported orthogonal wavelet. It is easy to compute but performs poorly in extracting high-frequency components due to its step-function structure. The Morlet wavelet, a continuous wavelet composed of a Gaussian-modulated sine wave, is suitable for high-resolution time–frequency analysis and has strong capability in extracting both high- and low-frequency information. However, it lacks orthogonality and incurs higher computational cost. The Daubechies wavelet is a discrete wavelet with orthogonality and compact support. Although it provides good time–frequency localization, it suffers from some loss of accuracy in decomposing both high- and low-frequency components. Therefore, the Coif3 wavelet is selected in this study for extracting high- and low-frequency information from spectrograms.

**Figure 3:**
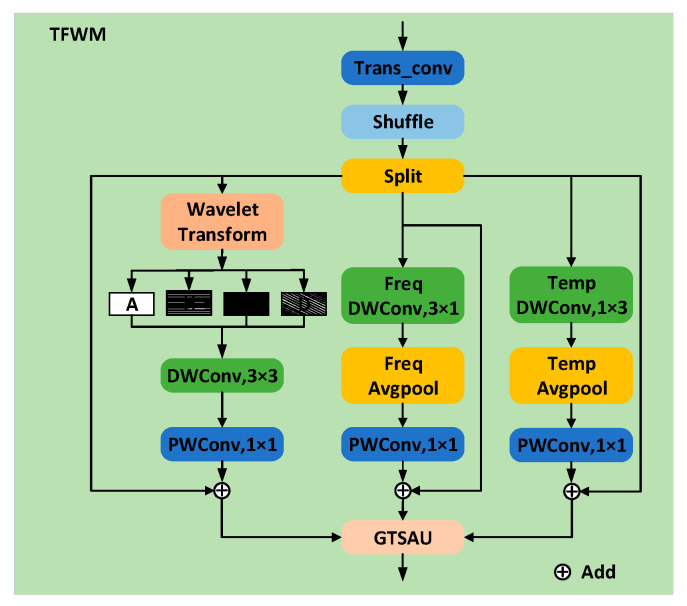
Time–frequency–wavelet module. Trans_conv: transposed convolution, Shuffle: channel shuffle, DWConv: depthwise convolution, PWConv: pointwise convolution, GTSAU: gated temporal–spatial attention unit, A: low-frequency component, H: horizontal high-frequency component, V: vertical high-frequency component, D: diagonal high-frequency component.

The time–frequency–wavelet module, shown in Figure 3, combines the high- and low-frequency information extracted via wavelet transform with a time–frequency separated convolutional path. Specifically, for the input feature map $X\in {\mathbb{R}}^{C\times H\times W}$, where C, H, and W denote the number of channels, height, and width of the feature map, respectively, after the shuffle unit performs the disruption operation on the channels, the feature map X is evenly divided into three parts in the channel dimension. Two of these parts are processed using one-dimensional (1D) convolutional kernels along the time and frequency dimensions, respectively, allowing the model to capture richer time–frequency features while reducing computational cost. The third part is decomposed using the Coif3 wavelet into a low-frequency component, a horizontal high-frequency component, a vertical high-frequency component, and a diagonal high-frequency component.

In this study, we borrow the Haar wavelet transform decomposition formula from the literature [25] but replace the Haar wavelet used therein with the Coif3 wavelet to extract more effective time–frequency features. The detailed procedure for extracting high- and low-frequency information from the spectrogram using the Coif3 wavelet is provided in Appendix A.

Time–frequency features are captured by applying the wavelet transform to extract the high- and low-frequency information of the spectrogram as a multi-channel input path, while maintaining the time and frequency paths. These features are then fed into the space–time gated attention unit module for feature fusion. This not only fully leverages the advantages of wavelet transform in extracting high- and low-frequency features but also further decouples and optimizes the features through time–frequency separated convolution, improving the performance and robustness of cross-device ASC.

### 2.3. Gated Temporal–Spatial Attention Unit

**Figure 4:**
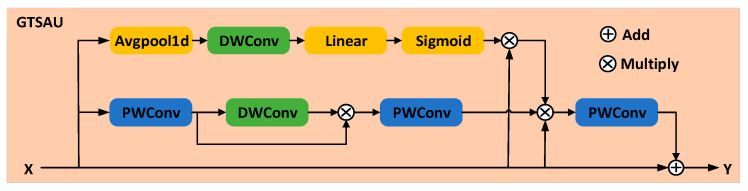
GTSAU module. DWConv: depthwise convolution, PWConv: pointwise convolution.

In the ASC task, the audio signal exhibits distinct characteristics and roles in the temporal and spatial dimensions. To effectively fuse multidimensional features and adaptively weight their contributions, this study integrates a temporal attention mechanism, a spatial attention mechanism, and a gated linear unit into the proposed GTSAU, whose structure is shown in Figure 4.

Specifically, in the GTSAU module, deep convolution is employed to reduce computational complexity. First, temporal and spatial attention mechanisms are applied to obtain time-weighted and spatial-weighted features, respectively. These weighted features are then element-wise multiplied with the input features to achieve feature fusion, followed by pointwise convolution to adjust the output dimensions. Finally, residual connections and learnable scaling parameters are introduced to enhance network stability and mitigate the gradient vanishing problem. The core computational process of the proposed GTSAU module is represented as follows:

(1) W=T×S×X,

(2) Y=α×W+X,

where X denotes the input feature map, T denotes the temporal feature attention weight, S denotes the spatial feature attention weight, W represents the fused result obtained from the interaction of temporal and spatial features, α is the learnable scaling parameter, and Y denotes the final output of the module, which combines the fused features and the input features through residual connection. With GTSAU, efficient and stable feature fusion is achieved, providing more robust audio representations for ASC tasks in complex acoustic environments.

### 2.4. VSS Module Based on Mamba Model

The Mamba model is a powerful framework built on the state space model (SSM). At its core, the SSM is a linear time-invariant system that maps one-dimensional functions or time-dependent input sequences to outputs via hidden states [26]. The Mamba model reduces the computational complexity of modeling long sequences from quadratic to linear [27]. The sequential nature of scanning operations in the Mamba model is highly consistent with natural language processing tasks involving time series data, but there are important challenges when applying it to audio data. Audio data not only exhibits temporal dependencies but also contains rich spectral information. To efficiently process this dual-domain information, a VSS module is introduced as a fundamental unit of the network [28].

**Figure 5:**
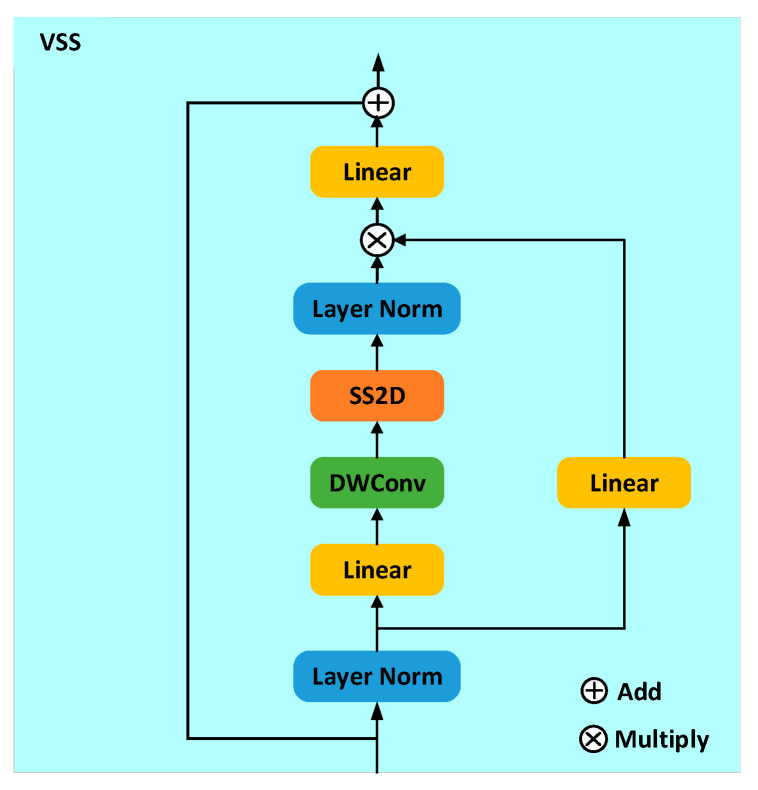
VSS module. DWConv: depthwise convolution, SS2D: 2D selective scan.

The VSS module forms four distinct sequences by using 2D Selective Scan (SS2D) to first expand the feature map along four directions [29]. Each feature sequence is then processed in parallel using a separate SSM block. Finally, these sequences are recombined into a complete, information-rich 2D feature map. The Selective Scanning Spatial State Sequence Model serves as the core SSM operator of the VSS block, enabling each element of a one-dimensional array to interact with all previously scanned elements through compressed hidden states. The overall structure of the VSS module is shown in Figure 5.

### 2.5. KAN Layers

To address the issues of low parameter efficiency and limited interpretability inherent in MLPs, KAN are based on the Kolmogorov–Arnold representation theorem, which states that any multivariate function can be represented as a composition of univariate continuous functions and additive operations. Essentially, a KAN is a hybrid of spline functions and MLPs, where the network replaces fixed activation functions at the nodes with learnable activation functions along the edges, enabling it to better approximate target functions. In the proposed TFWFNet model, a KAN layer is integrated into the classifier to enhance expressiveness and generalization. The mathematical formulation of the KAN layer is presented in Equation (3) [30]:

(3) $$KAN(Z)=\Phi_{K-1}\circ \Phi_{K-2}\circ \cdot \cdot \cdot \circ \Phi_{0}(Z),$$

where $\Phi_{i}$ denotes the function matrix corresponding to the i-th KAN layer (B-spline function matrix) and Z is the input matrix.

### 2.6. Dataset and Experimental Setup

The TAU Urban Acoustic Scenes 2022 Mobile development dataset is a publicly available dataset provided by the DCASE website. It consists of a training subset and a validation subset [31]. The development dataset includes recordings from 10 cities and 9 devices, comprising 3 real devices (A, B, and C) and 6 simulated devices (S1–S6). The total duration of the development dataset is 64 h, comprising 230,350 audio clips. Each audio clip is 1 s long, sampled at 44.1 kHz, and stored in WAV format. The DCASE official website provides five training subsets, corresponding to 5%, 10%, 25%, 50%, and 100% of the full development dataset. Table 1 lists the number of audio clips included in each training subset.

The UrbanSound8K dataset is a publicly available dataset [32]. It contains 10 categories of urban sounds: air conditioners, car horns, children playing, dogs barking, drilling, idling engines, gunshots, power drills, sirens, and street music. The dataset consists of a total of 8732 audio clips, each labeled with one of the 10 categories. The duration of each audio clip ranges from 0 to 4 s.

In the audio preprocessing stage, each audio clip is first resampled to 32 kHz and the spectrogram is extracted using a 4096-point FFT with a window size of 96 ms and a frame shift of 16 ms. The spectrogram is then converted to a Mel spectrogram using a bank of 512 Mel filters.

The device information used to train the network is as follows: Manufacturer: Lianzhong Cluster (Beijing) Technology Co., Ltd., Beijing, China; System: Ubuntu 20.04.6 LTS; GPU: NVIDIA GeForce RTX 3090; CPU: Intel(R) Core(TM) i9-10900X CPU @ 3.70 GHz; CUDA: version 11.8. In the experiments, the batch size was set to 256, the learning rate was set to 0.001, and optimization was performed using the Adam optimizer. Different hyperparameters were set for different data augmentation methods. *α* for MixUp was set to 0.5. *α* and P for Freq MixStyle were set to 0.5 and 0.9, respectively. The mask ratio and P for SpecAugmentation were set to 0.2 and 0.9, respectively. P_DIR_ for DIR was set to 0.6.

## 3. Results

In this paper, a series of experiments were conducted on the TAU Urban Acoustic Scenes 2022 Mobile development dataset and the UrbanSound8k dataset. No pre-trained model was used in the experiments, which were designed to validate the performance of the TFWFNet model. Accuracy was used as a performance metric.

### 3.1. Classifier Selection

Table 2 presents a comparison between classifiers using MLPs and KAN layers on the TAU Urban Acoustic Scenes 2022 Mobile development dataset. The experimental results demonstrate that classifiers with KAN layers consistently achieve superior classification performance, attaining an average accuracy of 54.24% across different training subset sizes. Under the same experimental conditions, the accuracy of classifiers employing KAN layers on the test set improves by an average of 0.94 percentage points compared to traditional MLP classifiers. This performance advantage primarily stems from the unique structural properties of KAN layers; by introducing learnable activation functions, KAN layers more effectively capture complex nonlinear relationships in the data. Consequently, KAN layers are chosen as the classifier component in this study’s model, not only enhancing classification accuracy but also providing a solid architectural foundation for handling more complex classification tasks in the future. Additionally, the design of KAN layers offers flexible scalability for further optimization of classification performance.

### 3.2. Ablation Experiments

To further validate the effectiveness of each component, this paper conducts experiments by gradually adding individual modules to the TFWFNet model on the TAU Urban Acoustic Scenes 2022 Mobile development dataset, observing the impact of each module on model performance. Table 3 presents the classification accuracy of the model on each training subset after removing individual modules. The experimental results clearly show that each module contributes positively to the overall performance of the TFWFNet model. TFWConv + GTSAU + KAN achieve an average classification accuracy of 54.30%, demonstrating the improvement brought by the GTSAU module. This improvement is attributed to the GTSAU’s enhancement of global spatio-temporal adaptive capabilities, providing more robust audio features for ASC and improving the model’s adaptability to complex data. TFWConv + VSS + KAN achieve an average classification accuracy of 53.97% by capturing contextual information and spatial relationships through the VSS module. The complete TFWFNet model, which integrates all modules, achieves the highest average accuracy of 56.16%, indicating the best classification performance. This demonstrates that the model fully leverages the advantages of each module, significantly enhancing classification accuracy.

### 3.3. Comparison with Other Methods

In this paper, the classification accuracies are shown in Table 4 compared to other methods with the same dataset. The methods mentioned in Table 4 are as follows:

DECASE Baseline is a benchmark model officially provided by the DCASE Challenge for performance comparison of ASC tasks [33].

MofleNet57 is a variant of MofleNet (MobileShuffleNet) [34], which is a network architecture that combines channel shuffling and residual inverted bottleneck blocks with 57 layers. The network model introduces channel shuffling, which not only facilitates the capture of richer features by the network but also reduces the number of parameters without significantly affecting the information exchange between channels.

CP-ResNet59 is a variant of the CP-ResNet model [35], which maintains the depth of the network model while controlling the sensory field of the model by adjusting the size of the convolution kernel, which has a total of 59 layers, and this design helps the network model to extract both local and global information of the features.

BC-PACN-48 is an improvement of parallel attention–convolution network (PACN) [36], which replaces the local contextual information module inside the PACN network using broadcast residual blocks (BC-ResBlocks); BC-ResBlocks are used to extract the feature maps in frequency and time dimensions by 2D convolution in the frequency direction and 1D convolution in the time direction, respectively, so as to enhance the ability of time–frequency feature expression, and 48 means that the feature dimensions of the model are set to 48.

The TF-SepNet [16] network is used for feature extraction by using a 1D convolution kernel in the time and frequency dimensions, which reduces the computational cost while providing a larger sensory field, thus enabling the model to capture more audio features, and the TF-SepNet-64 model adapts the base channel of the TF-SepNet model to 64.

As shown in Table 4, the proposed method achieves the highest average accuracy of 56.16% on the TAU Urban Acoustic Scenes 2022 Mobile development dataset, significantly outperforming existing baseline and comparison models. An in-depth analysis of the experimental results reveals three key findings: First, across comparison experiments with varying training subset sizes, this method consistently outperforms the comparison models on the test set. Notably, even when only 5% of the training data is used, it maintains an accuracy of 47.84%, demonstrating excellent generalization ability in low-data scenarios. Second, compared to the DECASE Baseline method, the proposed method improves classification accuracy by 6.53% in complex urban acoustic environments. Third, the proposed method exhibits stronger robustness when handling audio data from multiple devices.

These results fully demonstrate the effectiveness of the proposed method in feature extraction and classification. This success is mainly attributed to three factors: (1) the TFWM proposed in this paper effectively extracts information from the spectrogram and captures variability across cross-device audio data, while the GTSAU module efficiently fuses these features; (2) the introduction of the VSS module enhances the network’s ability to model contextual information; and (3) incorporating KAN layers as classifiers improves the model’s generalization capability. Overall, the proposed method not only enhances model performance but also offers a more reliable solution for ASC tasks in real-world applications.

**Figure 6:**
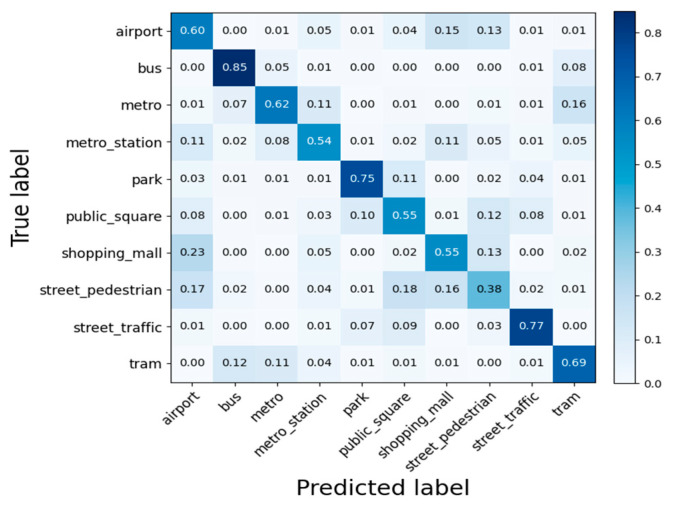
Confusion matrix for test set on 100% development subset.

The confusion matrix of the test set on the 100% development subset is shown in Figure 6. From the results, the accuracy gap between bus and street pedestrian classes is large, mainly due to the similarity, diversity, and complexity of the sounds, which can easily cause confusion in scene categorization.

### 3.4. Generalization Experiments

To verify that the proposed TFWFNet model also demonstrates superior classification ability on other datasets, its effectiveness is evaluated on the UrbanSound8K dataset. The model’s performance is compared with existing algorithms, including Efficient Residual Audio Neural Networks (ERANNs) [37], Deep Convolutional Neural Networks with regularization and data augmentation (DCNN + regularization + augmentation) [38], GoogLeNet [39], and using pre-trained VGGish [40], as shown in Table 5.

As shown in Table 5, the TFWFNet model achieved a classification accuracy of 97.60% without using pre-trained weights and was trained from scratch, surpassing the 96.70% accuracy of the pre-trained VGGish-based model and achieving the best classification performance among all compared methods. This demonstrates that the model is better able to capture key information in the audio signal for the ASC task. The model’s performance on the UrbanSound8K dataset further validates its effectiveness. In conclusion, the TFWFNet model demonstrates great potential in ASC tasks and offers new insights for research in related fields.

**Figure 7:**
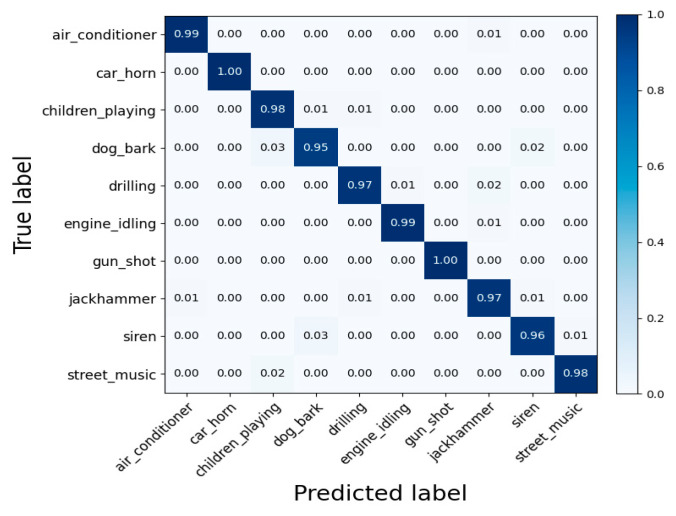
UrbanSound8K dataset confusion matrix.

In this study, the confusion matrix of the UrbanSound8K dataset using the TFWFNet model is presented in Figure 7. The results show that the classification accuracy of the TFWFNet model exceeds 95% across all audio categories, with car_horn and gun_shot achieving 100% accuracy. The lowest accuracy is observed for dog_bark, likely due to the similarity of its background sounds or spectral features with those of other audio classes.

## 4. Discussion

The TFWFNet model proposed in this study has achieved significant results in ASC. It improves the recognition accuracy of audio scenes and provides strong technical support for intelligent systems to implement environment-aware capabilities.

In order to further mine the information in the spectrogram, this study optimizes the basic structure of the TF-SepNet model so as to achieve improvement in accuracy; the wavelet transform is introduced into the dual-path structure of time and frequency dimensions of the TF-SepNet model to extract the high- and low-frequency information paths of the spectrogram, and the information from the three paths is efficiently fused by the proposed GTSAU module to provide richer feature information for the model. The VSS module is also introduced to capture the audio data contextual information and spatial relationships. Finally, the KAN layers are adopted as a classifier, which can enhance the nonlinear representation of the model.

In this process, this study was validated by ablation comparison test. As shown in Table 2, the average accuracy of the model increased by 0.94% after using the KAN layer instead of the MLPs, which facilitates the model to learn different levels of feature information more deeply in the training stage. As indicated in Table 3, the most significant performance improvement of the model was achieved after the joint use of the GTSAU module and the VSS module. The model takes advantage of the strengths of each module, which facilitates the model to fully utilize the information in the spectrogram and achieve improvement in accuracy.

This investigation was conducted on the TAU Urban Acoustic Scenes 2022 Mobile development dataset and the UrbanSound8K dataset, which fully demonstrated the significant advantages of the proposed model in this study in terms of accuracy by comparing it with other methods. The specific data are shown in Table 4 and Table 5. The experimental data show that the TFWFNet network on the TAU Urban Acoustic Scenes 2022 Mobile development dataset improves the accuracy rate to 56.16% and, on the UrbanSound8K dataset, the accuracy rate reaches 97.60%.

When performing scene recognition on the TAU Urban Acoustic Scenes 2022 Mobile development dataset, as shown in Figure 6, it was observed that the recognition accuracy for street pedestrian scenes was relatively low. This is primarily because the sounds in this category are easily confused with those of other scenes. To address this limitation and enhance the recognition performance for this category, we plan to expand the dataset by including more diverse scene samples in future studies. Through this strategy, we aim to improve the overall accuracy and reliability of scene recognition across all categories.

However, there is still room for improvement in accuracy compared to other methods [41,42]. This is mainly attributed to limitations in the model’s feature learning capabilities, particularly its limited ability to distinguish between sounds in complex acoustic environments and its weak robustness to background noise. To enhance the model’s performance, several optimization strategies can be considered. On the one hand, incorporating a pre-trained audio model as a feature extractor can improve the model’s capacity to perceive complex acoustic features, thereby facilitating better learning and recognition of various scene categories. On the other hand, the dataset can be expanded by introducing audio samples from diverse environments and devices, which would enable the model to learn a more comprehensive distribution of acoustic features, thereby improving both recognition accuracy and generalization ability.

## 5. Conclusions

This paper proposes an ASC model based on a time–frequency–wavelet fusion network, which effectively enhances ASC performance by incorporating the TFWM, GTSAU module, VSS module, and KAN layers.

The TFWM module employs the Coif3 wavelet to extract high- and low-frequency information from spectrograms, addressing both local and global aspects of the signal while significantly enhancing the model’s ability to capture critical spectrogram features. The GTSAU module integrates temporal attention, spatial attention, and gated units to emphasize temporally and spatially salient features, effectively fusing the multi-path features extracted by the TFWM module. The VSS module strengthens the model’s capacity to capture contextual information and spatial relationships within audio data. Finally, as the classifier, the KAN layers employ learnable activation functions to model complex information, thereby enhancing the generalization capability of the TFWFNet model.

The experiments were conducted on the TAU Urban Acoustic Scenes 2022 Mobile development dataset and the UrbanSound8K dataset to validate the effectiveness of the model. The results show that the model achieves accuracies of 47.84%, 51.88%, 57.89%, 60.06%, and 63.14% on the 5%, 10%, 25%, 50%, and 100% training subsets, respectively, with an average accuracy of 56.16%. On the UrbanSound8K dataset, the accuracy reaches 97.60%, fully demonstrating the model’s effectiveness and robustness. In summary, the time–frequency–wavelet fusion network-based acoustic scene classification model proposed in this paper performs well and provides a promising new solution for research in this field.

## Figures and Tables

**Table 1:** TAU Urban Acoustic Scenes 2022 Mobile development dataset and number of individual development training subsets [31].

Devices Name	Type	100% Subset	50% Subset	25% Subset	10% Subset	5% Subset
A	Real	102,150	51,100	25,520	10,190	5080
B	Real	7490	3780	1900	730	380
C	Real	7480	3780	1920	790	380
S1	Simulated	7500	3720	1840	740	380
S2	Simulated	7500	3700	1850	750	380
S3	Simulated	7500	3720	1870	760	380
Total		139,620	69,800	34,900	13,960	6980

**Table 2:** Classifier selection.

Model	5% Subset	10% Subset	25% Subset	50% Subset	100% Subset	Avg
TFWConv + MLPs	44.36	48.52	54.67	58.44	60.54	53.30
TFWConv + KAN layers	45.68	49.48	55.38	58.86	61.83	54.24

**Table 3:** Ablation experiments with the TFWFNet model.

Model	5% Subset	10% Subset	25% Subset	50% Subset	100% Subset	Avg
TFWConv + GTSAU + KAN layers	44.61	50.22	55.66	60.83	60.21	54.30
TFWConv + VSS + KAN layers	45.22	50.05	55.77	59.12	61.97	54.43
ours	47.84	51.88	57.89	60.06	63.14	56.16

**Table 4:** Comparison of models.

Model	5% Subset	10% Subset	25% Subset	50% Subset	100% Subset	Avg
DECASE Baseline	42.40	45.29	50.29	53.19	56.99	49.63
MofleNet57	41.64	45.46	52.46	56.85	59.31	51.14
CP-ResNet59	44.98	49.28	54.93	57.87	59.57	53.33
BC-PACN-48	46.44	52.61	57.41	59.75	61.95	55.63
TF-SepNet-64	45.70	51.10	55.60	59.60	62.50	54.90
ours	47.84	51.88	57.89	60.06	63.14	56.16

**Table 5:** Accuracy of classification for UrbanSound8K dataset.

Model	Accuracy
ERANNs [37]	90.80%
GoogLeNet [39]	93.00%
pre-trained VGGish [40]	96.70%
ours	97.60%

## Data Availability

The data supporting the findings of this study are available upon reasonable request.

## Abbreviations

The following abbreviations are used in this manuscript:

ASC	Acoustic scene classification
DCASE	Detection and Classification of Acoustic Scenes and Events
SSM	State space model
AuM	Audio Mamba
AST	Audio Spectrum Transformer
MLPs	Multilayer Perceptrons
KAN	Kolmogorov–Arnold Network
HPSS	Harmonic-Shock Source Separation
CNN	Convolutional Neural Network
TF-SepNet	Time–Frequency Separate Network
TFWFNet	Time–Frequency–Wavelet Fusion Network
TFWM	Time–Frequency–Wavelet Module
GTSAU	Gated Temporal–Spatial Attention Unit
VSS	Visual State Space
DWConv	Depthwise convolution
PWConv	Pointwise Convolution
ResNorm	Residual Normalization
Trans_conv	Transposed Convolution
Shuffle	Channel Shuffle
SS2D	2D Selective Scan
DIR	Device Impulse Response
Coif3 Wavelet	Coiflet Wavelet Family, Order 3
PACN	Parallel Attention–convolution Network
BC-ResBlocks	Broadcast residual blocks
ERANNs	Efficient Residual Audio Neural Networks
DCNN	Deep Convolutional Neural Networks

## Appendix A

The low-pass filter coefficients $h_{k}$ and the high-pass filter coefficients $g_{k}$ of the Coif3 wavelet are shown in Equations (A1) and (A2).

(A1) $$h_{k}=\left[\begin{array}{ccc} -3.459977319727278\times 10^{-5}, & -7.0983302506379\times 10^{-5}, & 0.0004662169598204029,\\ 0.0011175187708306303, & -0.0025745176881367972, & -0.009007976136730624,\\ 0.015880544863669452, & 0.03455502757329774, & -0.08230192710629983,\\ -0.07179982161915484, & 0.42848347637737, & 0.7937772226260872,\\ 0.40517690240911824, & -0.06112339000297255, & -0.06577191128146936,\\ 0.023452696142077168, & 0.007782596425672746, & -0.003793512864380802 \end{array}\right],$$

(A2) $$g_{k}=\left[\begin{array}{ccc} 0.003793512864380802, & 0.007782596425672746, & -0.023452696142077168,\\ -0.06577191128146936, & 0.06112339000297255, & 0.40517690240911824,\\ -0.7937772226260872, & 0.42848347637737, & 0.07179982161915484,\\ -0.08230192710629983, & -0.03455502757329774, & 0.015880544863669452,\\ 0.009007976136730624, & -0.0025745176881367972, & -0.0011175187708306303,\\ 0.0004662169598204029, & 7.0983302506379\times 10^{-5}, & -3.459977319727278\times 10^{-5} \end{array}\right],$$

For single-channel feature map $X_{C}\in {\mathbb{R}}^{H\times W}$, where H and W denote the height and width of the single-channel feature map, the first-order Coif3 wavelet transform is computed as shown in Equation (A3) through Equation (A8):

First, a one-dimensional decomposition of the feature map in the row direction is performed:

(A3) $$A_{c}(i,j)=\sum_{k}h_{k}X_{c}(i,2j-k),$$

(A4) $$D_{c}(i,j)=\sum_{k}g_{k}X_{c}(i,2j-k),$$

where i∈[0,H−1] denotes the row index, $j\in [0,\frac{W-1}{2}]$ denotes the column index after downsampling, and k denotes the length of the filter (k=0,1,…,18). $A_{c}(i,j)$ is the low-frequency approximation coefficient for channel C and $D_{c}(i,j)$ is the high-frequency detail coefficient for channel C.

Then, the low-frequency approximation coefficient $A_{c}(i,j)$ and the high-frequency detail coefficients $D_{c}(i,j)$ are further decomposed in the column direction:

(A5) $$A_{c}=\sum_{k}h_{k}A_{c}(2i-k,j),$$

(A6) $$H_{c}=\sum_{k}g_{k}A_{c}(2i-k,j),$$

(A7) $$V_{c}=\sum_{k}h_{k}D_{c}(2i-k,j),$$

(A8) $$D_{c}=\sum_{k}g_{k}D_{c}(2i-k,j),$$

where $i\in [0,\frac{H-1}{2}]$ denotes the row index after downsampling and $j\in [0,\frac{W-1}{2}]$ denotes the column index. $A_{c}$ denotes the low-frequency approximation coefficients for a single channel, $H_{c}$ denotes the high-frequency detail coefficients for a single channel in the horizontal direction, $V_{c}$ denotes the high-frequency detail coefficients for a single channel in the vertical direction, and $D_{c}$ denotes the high-frequency detail coefficients for a single channel in the diagonal direction. Finally, the four components are spliced in the channel dimension to form a multichannel input.
